# Light Variability Illuminates Niche-Partitioning among Marine Picocyanobacteria

**DOI:** 10.1371/journal.pone.0001341

**Published:** 2007-12-19

**Authors:** Christophe Six, Zoe V. Finkel, Andrew J. Irwin, Douglas A. Campbell

**Affiliations:** Mount Allison University, Sackville, New Brunswick, Canada; Monash University, Australia

## Abstract

*Prochlorococcus* and *Synechococcus* picocyanobacteria are dominant contributors to marine primary production over large areas of the ocean. Phytoplankton cells are entrained in the water column and are thus often exposed to rapid changes in irradiance within the upper mixed layer of the ocean. An upward fluctuation in irradiance can result in photosystem II photoinactivation exceeding counteracting repair rates through protein turnover, thereby leading to net photoinhibition of primary productivity, and potentially cell death. Here we show that the effective cross-section for photosystem II photoinactivation is conserved across the picocyanobacteria, but that their photosystem II repair capacity and protein-specific photosystem II light capture are negatively correlated and vary widely across the strains. The differences in repair rate correspond to the light and nutrient conditions that characterize the site of origin of the *Prochlorococcus* and *Synechococcus* isolates, and determine the upward fluctuation in irradiance they can tolerate, indicating that photoinhibition due to transient high-light exposure influences their distribution in the ocean.

## Introduction

The smallest category of free living photosynthetic cells is picophytoplankton, defined as less than 3 µm diameter. Picophytoplankton cells, although individually minute, dominate carbon assimilation and primary productivity over large areas of the ocean. Among the taxonomically diverse groups composing the picophytoplankton the cyanobacteria *Synechococcus* and *Prochlorococcus* are major contributors to primary production and carbon export over large areas of the open ocean [1]. *Prochlorococcus*, the most abundant photosynthetic organism on Earth [2], is restricted to the warm rather oligotrophic waters of the latitudinal band extending from 40°N to 40°S [3]–[5] and laboratory experiments show it does not grow well at low temperatures [6]. *Synechococcus* and *Prochlorococcus* co-occur in many oceanographic regions, but *Synechococcus* tolerates a broader temperature range [6], [7] and thrives in more meso- and eutrophic waters, even though *Prochlorococcus* can also grow at these higher nutrient levels [2]. *Synechococcus* are often less abundant in warmer, oligotrophic ecosystems where *Prochlorococcus* is the major primary producer [2], [5].

*Prochlorococcus* and *Synechococcus* have cell types (often referred to as ecotypes) which have identifiable geographic ranges that correspond to particular temperature, nutrient concentration, as well as light regimes [2]. *Synechococcus* cell types differ in their pigment content, allowing these organisms to exploit specific spectral niches [8]–[10], which tend to vary along a horizontal offshore-onshore axis within the upper mixed layer [11]–[15]. In contrast, *Prochlorococcus* ecotypes are found at different depths in the water column, and are adapted to different average irradiance [2], . The surface ecotypes of *Prochlorococcus* have optimal growth irradiances similar to *Synechococcus* strains [6], [19], [20]. Average irradiance contributes to niche partitioning with depth among *Prochlorococcus* ecotypes, but even in combination with temperature and nutrient regime, does not fully account for the differential distribution of the *Prochlorococcus* and the *Synechococcus* strains. In particular, the absence of *Prochlorococcus* in temperate, permanently mixed shallow seas such as the English Channel where *Synechococcus* is very abundant, remains poorly understood [2].

The ocean is a dynamic environment in which phytoplankton must cope with rapid changes in resources, particularly irradiance [21], [22]. For a phytoplankton cell, irradiance changes rapidly if light attenuation and mixing in the water column are large, as the cell moves vertically through a large depth/irradiance gradient. Downward mixing of a phytoplankton cell leads to lower irradiance and therefore a decrease in growth, but with no immediate risk of cellular death. In contrast, when a cell is taken upwards in the water column, it must often withstand both rapid and large increases in irradiance. To maintain photosynthesis and viability, phytoplankton must counter the photoinactivation of photosystem II (PSII) [23], [24] with repair [25] through proteolytic removal of photodamaged D1 protein [26] and the coordinated insertion of newly synthesized D1 into the thylakoid membrane [27]. If an increase in irradiance causes photoinactivation to outrun repair, the cell suffers net photoinhibitory loss of photosynthetic capacity, leading potentially to cell death. The risk of exposure to upward fluctuations in irradiance may therefore constitute a potent selective pressure contributing to niche partitioning among cyanobacterial cell types.

To determine if upward fluctuations in irradiance are an important selective factor in niche partitioning among marine picocyanobacteria, we quantitatively analyzed the relative capacities to tolerate a sudden increase in irradiance across five ecologically significant types of *Synechococcus* and *Prochlorococcus* isolated from habitats with contrasting dynamic irradiance regimes.

## Results and Discussion

**Figure 1:**
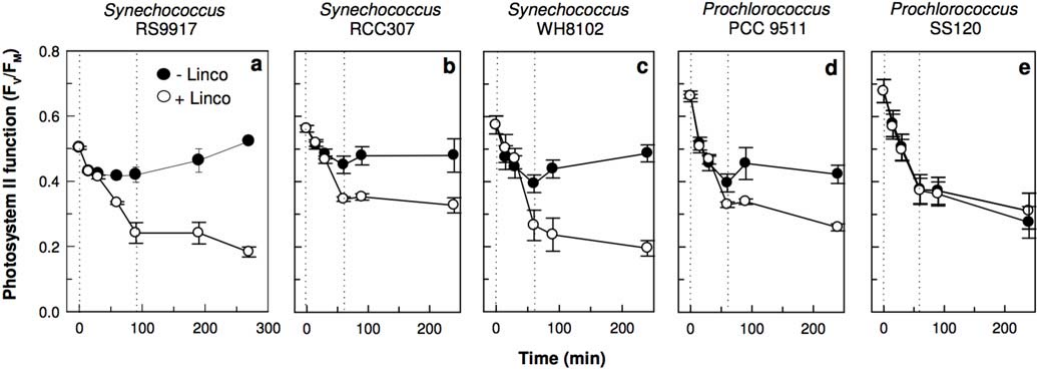
Five marine cyanobacteria from a range of ecological niches show distinct responses of photosystem II quantum yield (F_V_/F_M_), reflecting photosystem II activity, to a 10 fold irradiance increase episode followed by recovery under growth light. The high light episode is delineated by the dotted lines. Cultures were treated (closed) or not (open) with the protein synthesis inhibitor lincomycin to block photosystem II repair (n = 4, ±1 s.e.). Note the strong recovery of photosystem II function in *Synechococcus* sp. RSS9917, and the lack of recovery in *Prochlorococcus* sp. SS120.

The *Synechococcus* and *Prochlorococcus* cell types exhibited a gradient in their photophysiological tolerance of upward fluctuations in irradiance (Fig. 1), resulting from different capacities to induce repair (*R_PSII_*, functional PSII gained s^−1^) to counter the PSII photoinactivation rate (PSII lost s^−1^). To tolerate and therefore exploit upward fluctuations in irradiance, PSII repair must equal the magnitude of the rate of PSII photoinactivation, which we parameterized as:
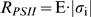
(1)where E is the scalar irradiance in photons nm^−2^ s^−1^ and σ_i_ is the effective target size for photons driving PSII photoinactivation [28], with nominal units of nm^2^. If *R_PSII_*<E•|σ_i_|, the cells suffer a net loss of photosynthetic capacity termed photoinhibition [27], and eventually cell death. Quantifying the parameters in Eq. (1) allowed us to determine the basis for different capacities among the *Synechococcus* and *Prochlorococcus* cell types to cope with upward fluctuations in irradiance, thereby illuminating their distributions in the ocean.

**Figure 2:**
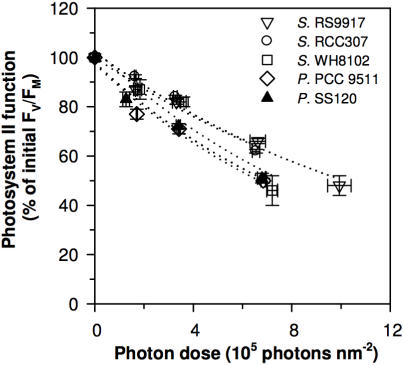
Five marine cyanobacteria show comparable inhibition of Photosystem II plotted versus cumulative photon dose (µmol photons nm^−2^ s^−1^×s), when photosystem II repair is blocked (lincomycin treated cultures; n = 4, ±1 s.e.). Open triangle: *Synechococcus* RS9917; open circle: *Synechococcus* RCC307; open square: *Synechococcus* WH8102; open diamond: *Prochlorococcus* PCC 9511; closed triangle: *Prochlorococcus* SS120.

We estimated σ_i_, the effective target size for photons driving PSII photoinactivation under blue light (see Supplementary Data S1 and Figure S1 for the choice of parameterization through target theory), as the exponential decay of PSII function plotted versus cumulative photon dose nm^−2^ (Fig. 2). We separated the primary photoinactivation of PSII from the counteracting repair using lincomycin, an inhibitor of 16S ribosomal function, to block the synthesis of the D1 protein, thus preventing any PSII repair (Fig. 1). We then monitored the PSII activity by fluorimetry. When *R_PSII_* was blocked, σ_i_ fell in a narrow range across the five strains (Table 1; Fig. 2), with an average magnitude of 9.1×10^−7^±0.7×10^−7^ nm^2^, comparable to earlier estimates for the photoinactivation target size for higher plants [28]. For a given irradiance wavelength range, σ_i_ is likely a fundamental parameter of PSII across oxygenic photosynthetic organisms and growth conditions. In contrast the functional antenna size driving PSII photochemistry (σ_PSII_) varied widely among the strains (Table 1). In blue light, σ_PSII_ is ∼2–3×10^6^ times larger than the magnitude of σ_i_ and the ratio σ_PSII_/|σ_i_| estimates the relative probability of PSII photochemistry versus PSII photoinactivation. Our results are consistent with PSII photoinactivation depending upon a rare, rate-limiting initial photon capture by a target separate from the main photosynthetic antenna, probably within the oxygen evolving subcomplex of PSII [27], [29], [30]; (see Supplementary Data S1, Figure S1).

**Table 1:** Origins and photophysiological features of the five marine cyanobacteria used in this study.

	*Synechococcus* RS9917	*Synechococcus* RCC307	*Synechococcus* WH8102	*Prochlorococcus* PCC 9511	*Prochlorococcus* SS120
Origin	Gulf of Aqaba, surface	Mediterranean, surface	Caribbean Sea, surface	Sargasso Sea, surface	Sargasso Sea, 120 m depth
Water regime	Eutrophic	Mesotrophic	Oligotrophic	Oligotrophic	Oligotrophic
Antenna type	Small PBS, A_max_∼620 nm	Large PBS, A_max_∼550 nm	Large PBS, A_max_∼495 nm	Pcb ring, A_max_∼465 nm	Pcb ring, A_max_∼465 nm
NPQ	0.07±0.02	0.43±0.16	0.22±0.01	0.07±0.02	0.04±0.01
σ_PSII_ (nm^2^ PSII^−1^)	0.2±0.02	1.7±0.3	2.8±0.1	2.1±0.1	2.9±0.5
D1 content (fmol µg protein^−1^)	34±5	21±2	26±6	78±7	102±15
Protein specific σ_PSII_ (nm^2^ µg protein^−1^)	0.5±0.01×10^10^	2.1±0.04×10^10^	4.3±0.04×10^10^	9.8±0.04×10^10^	18±0.5×10^10^
|σ_i_| (nm^2^)	7.6±0.7×10^−7^	7.4±0.3×10^−7^	11±0.02×10^−7^	9.7±0.4×10^−7^	9.5±0.7×10^−7^
*R_PSII_* (s^−1^)	1.3±0.2×10^−4^	1.1±0.1×10^−4^	1.6±0.4×10^−4^	0.9±0.3×10^−4^	0.1±0.05×10^−4^
*E*_TOL_l, (µmol m^−2^ s^−1^)	283±26	255±5	223±31	152±45	20±9

PBS, phycobilisome; Pcb, Prochlorophyte chlorophyll binding protein; NPQ, non photochemical quenching of fluorescence induced at 300 µmol photons m^−2^ s^−1^; σ_PSII_, PSII effective absorbance cross section for blue light; |σ_I_|, magnitude of the effective target size for PSII photoinactivation by blue light; *R_PSII_*, PSII repair rate; *E*_TOL_, maximal variable irradiance (n = 4±s.e.).

In spite of their comparable σ_i_, these picocyanobacteria showed different tolerances to a sudden onset of high irradiance, which were largely explicable through differences in their inducible *R_PSII_* (Table 1). The *Synechococcus* strains all rapidly induced a strong *R_PSII_* in response to increased irradiance, thereby countering the increased photoinactivation rate and limiting any net decrease in PSII capacity. The same induction of *R_PSII_* under high irradiance supported rapid subsequent recovery of PSII capacity upon a return to low irradiance, particularly in the coastal *Synechococcus* RS9917 and the mesotroph *Synechococcus* RCC307 (Fig. 1A, B). The *Prochlorococcus* strains are functionally differentiated from the *Synechococcus* by their weaker inducible *R_PSII_*, especially in the low light adapted *Prochlorococcus* SS120, which showed negligible induction of *R_PSII_* in response to transient high light exposure (Table 1), and no ability to recover within 3 h of a return to low light (Fig. 1E). Only two of the *Synechococcus* strains induced a modest non-photochemical quenching to divert excitation from reaction centre II [31], [32] (Table 1), and in all strains the recovery from high irradiance was thus dependent upon protein synthesis (Fig. 1, Fig. S2), and not upon relaxation of non-photochemical quenching of fluorescence.

We compared the tolerance of the strains of a short-term increase in irradiance by estimating the maximum irradiance, *E*_TOL_, at which rapidly inducible repair can counter photoinactivation for each strain through a rearrangement of Eq. (1): *E*_TOL_ = *R_PSII_*/|σ_i_|. The coastal *Synechococcus* RS9917 could withstand a remarkable 14-fold short-term increase above its acclimated low growth irradiance through rapid induction of *R_PSII_* to counter the increased rate of photoinactivation (Table 1). This ability to exploit upward fluctuations in irradiances decreases among the strains from onshore to deep offshore waters (Table 1). The deep-sea ecotype *Prochlorococcus* SS120 showed little capacity to withstand a short-term exposure to an upward fluctuation in irradiance (Table 1), and no capacity for subsequent recovery within 3 h (Fig. 1), in keeping with selection for a deep ecological niche characterized by low and stable irradiance. Both *Prochlorococcus* strains contain significantly more of the PSII D1 protein (Table 1, Figure S2) than do the *Synechococcus* strains. Maintaining this heavy investment may be untenable for *Prochlorococcus* in the face of faster PSII photoinactivation under increased light. Moreover, *Prochlorococcus* possess large light harvesting antennae composed of membrane-intrinsic Prochlorophyte chlorophyll binding (Pcb) proteins [17], which form an annular ring around PSII [16]. We hypothesize that this Pcb antenna may hinder the turnover of photoinactivated D1 proteins (Figure S2), thereby limiting *Prochlorococcus* modulation of *R_PSII_* in comparison to the *Synechococcus* strains with extrinsic phycobilisome antennae.

**Figure 3:**
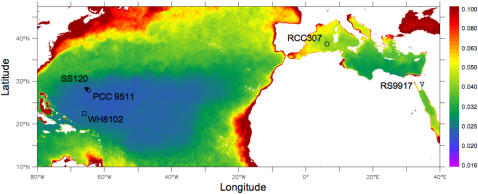
The ability of five marine cyanobacteria strains to tolerate short-term increases in irradiance (*E*_TOL_) relates to the vertical light attenuation coefficient (k_490_) at their location of origin. Color bar indicates the 2006 annual average vertical attenuation coefficient at 490 nm, k_490_. Symbols indicate the origin of the strains sampled near the surface (open symbols) except for SS120 strain sampled at 120 meters (closed triangle).

The abilities of these picocyanobacteria to withstand and exploit short-term exposure to high irradiance correlate with the origins of the strains along an onshore to offshore axis (Fig. 3). Coastal phytoplankton experience more variability in irradiance compared to open ocean organisms, notably due to an increase in the vertical attenuation of irradiance (k_d_) and water mixing in the water column towards shore (Fig. 3; [21], [22]. Vertical irradiance profiles near-shore change more rapidly with depth than in offshore waters. As a result, phytoplankton circulating in the near-shore water column experiences more rapid changes in irradiance under otherwise comparable conditions [21], [22]. The capacity for tolerance and exploitation of sudden irradiance changes thus appears less important in offshore, clear, stratified waters.

**Figure 4:**
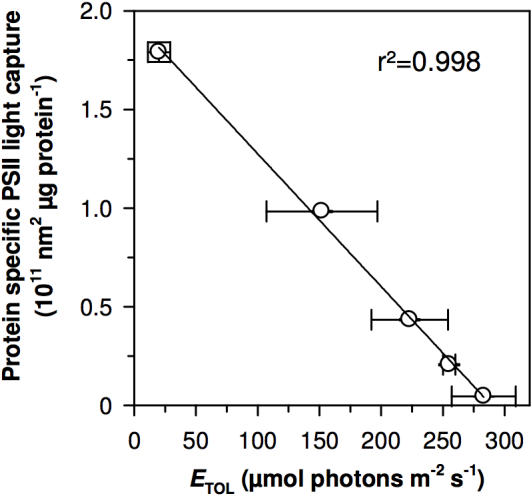
A trade-off between protein specific light capture capacity (protein specific σ_PSII_) and tolerance of irradiance variations (*E*_TOL_) across five marine cyanobacteria. *Prochlorococcus* strains show a high protein specific σ_PSII_, which varied 40-fold across the strains and shows a strong negative correlation with *E*_TOL_ (µmol photons m^−2^ s^−1^), the capacity to tolerate upward irradiance fluctuations, which was highest in the coastal *Synechococcus* RSS9917 (n = 4, ±1 s.e.; r^2^ = 0.98).

*Prochlorococcus* cells dominate over *Synechococcus* of the WH8102 type in oligotrophic marine ecosystems [2], [5], even though *Synechococcus* WH8102 shows comparable functional photosynthetic antenna size per PSII (Table 1) and a higher capacity to tolerate and exploit upward fluctuations in irradiance. The large phycobilisome of *Synechococcus* WH8102 is, however, more expensive in nitrogen than the Pcb antenna of *Prochlorococcus* [33]. Despite the superior ability of *Synechococcus* WH8102 to exploit and recover from irradiance fluctuations the high nitrogen cost for its antenna may relegate this cell type to minority status in oligotrophic cyanobacterial communities. We find that the *Prochlorococcus* strains do achieve much higher capacity for PSII light capture per cellular protein investment, when compared to *Synechococcus* (Table 1; Fig. 4). Across the strains, protein-specific blue light capture capacity varied 40-fold, and showed a strong negative correlation with *E*_TOL_, the capacity to tolerate upward irradiance fluctuations (Fig. 4). The evolution from a *Synechococcus*-like ancestor to *Prochlorococcus* with a lower nitrogen cost Pcb photosynthetic antenna may have led to limitations on the induction of PSII repair, and a consequent susceptibility to irradiance fluctuations through specialization for stable, oligotrophic environments [33]. A constrained nitrogen budget may thus force a cellular allocation of resources between PSII repair capacity, altering *E*_TOL_, and the ability of cells to harvest light. *Prochlorococcus* may thus dominate these oligotrophic, stratified environments not only because of the relatively low nitrogen cost of their photosynthetic antennae but also because their limited modulation of PSII repair is feasible where there is little fluctuation in light.

Our measurements of the effective target cross-section for photosystem II photoinactivation show that this parameter is conserved across marine picocyanobacteria, likely as a fundamental property of photosystem II [28]. This σ_i_ can now be combined with active fluorimetry to efficiently estimate photosystem II repair rates and the maximum short-term increase in irradiance (*E*_TOL_) that can be tolerated and exploited by phytoplankton species or communities in the field. These parameters are therefore valuable components for future biogeochemical and ecosystem models of the distribution and abundance of picocyanobacteria, definitions of phytoplankton functional groups, and their responses to environmental change. Current models of picophotoautotroph community responses to environmental change have heretofore considered steady state parameters determined on fully acclimated cultures, including the optimal irradiance for growth (see e.g. [34]). We show here that surface *Prochlorococcus* have less capacity to induce PSII repair than marine *Synechococcus*, despite a similar optimal irradiance for growth [6], [19], [20] consistent with their geographic distribution. A high optimal irradiance for acclimated growth may not necessarily correlate with tolerance and exploitation of sudden irradiance increases, a dynamic factor contributing to niche-partitioning among marine picocyanobacteria.

## Materials and Methods

### Culturing and time course experiment

The marine cyanobacteria *Synechococcus* strains RS9917, WH8102, RCC307 [35] and *Prochlorococcus* strains PCC 9511 and SS120 [6], [18] were grown in PCR-S11 medium [36] in polystyrene culture flasks at 22°C and 25 µmol photons m^−2^ s^−1^ white light. These picocyanobacteria were selected because of their importance as representatives of the major ecological functional groups of marine picophytoplankton, because their genomes are sequenced and they are thus emerging model organisms, and because their small cell size and simple, consistent optical properties [37] facilitated the fluorescence measurements and estimates of effective absorbance cross sections.

Exponential cultures were split into two flasks. One was supplemented with 500 µg mL^−1^ lincomycin and both flasks were incubated in the dark for 10 min, to allow the antibiotic to penetrate the cells and inhibit ribosome function. The two flasks were then shifted for 60–90 min to ca. 280 µmol photons m^−2^ s^−1^ blue light (LEE Filter #183, Panavision; 455–479 nm peak transmission, 406–529 nm half-height width). Samples were collected at 15, 30 and 60 (and 90) min to measure biophysical properties and for later protein immunodetection. The sub-cultures were then shifted back to their initial growth light and sampled after 30 and 180 min of recovery.

### Fluorescence measurements

Culture aliquots were dark-adapted and a blue-green modulated measuring light (4 Hz; Xenon-PAM, Walz, Effetrich, Germany) was activated to measure F_0_. Actinic irradiance was then activated at 280 µmol photons m^−2^ s^−1^; after signal stabilisation (Ft level), a saturating light pulse (4,000 µmol photons m^−2^ s^−1^, 500 ms) was triggered to determine the light acclimated maximal fluorescence (F_M_′). The PSII inhibitor 3-(3,4-dichlorophenyl)-1,1-dimethylurea was then added and after signal stabilisation, a light pulse was triggered again to determine the maximal fluorescence F_M_ to estimate the photochemical yield of PSII, F_V_/F_M_ = (F_M_−F_0_)/F_M_ and NPQ = (F_M_−F_M_′)/F_M_′ under the treatment light level.

The light-acclimated effective absorption cross-section serving PSII photochemistry (σ_PSII_, nm^2^ PSII^−1^), reflecting the functional antenna size, was determined on a culture aliquot illuminated for 2 min under the treatment light level (blue LED, 455±20 nm), followed by a saturating single turn-over flash (blue LED, 455±20 nm; FIRe fluorimeter, Satlantic, Halifax, NS Canada) to determine the σ_PSII_ of the open PSII reaction center [38], [39]. We estimated the capacity for PSII light capture per cellular protein investment as the product of σ_PSII_ (nm^2 ^PSII^−1^) and D1 per µg protein (see below), assuming that under acclimation to low growth light, D1 protein content closely approximates functional PSII content [40].

For comparison with σ_PSII_, and to facilitate future modelling efforts, we chose to estimate an effective target cross section for PSII photoinactivation (σ_i_, nm^2^) by plotting the exponential decay of the PSII quantum yield F_V_/F_M_ in the absence of repair versus the cumulative photon dose nm^−2^ (see Supplementary Data S1 and Figure S1 for justification). Note that the σ_i_ and σ_PSII_ estimates are for blue irradiance, approximating the spectral light quality in marine environments. Under other wavelength ranges σ_i_ would differ because the absorbance cross section for photoinactivation is dependent upon wavelength [29], [41].

### Immunodetections

Cells were harvested on glass fibre filters (25 mm, Whatman) and the proteins were extracted by 3 thawing/sonicating rounds in extraction buffer. The total protein concentration was determined (Lowry protein assay kit, Biorad). Two µg of total protein were loaded on a 4–12% acrylamide precast NuPAGE gel (Invitrogen). Along with the samples, D1 protein standards (Agrisera) were loaded to establish a standard curve. Electrophoresis was run for 40 min at 200 V and the proteins were transferred to a PVDF membrane. Following the transfer, the membrane was immersed in blocking solution (Amersham Biosciences) for at least 2 hours. The PVDF membranes were successively incubated with primary antibodies directed against D1 (Agrisera, 1/50,000) in Tween-TBS in the presence of 2% blocking agent and anti-chicken secondary antibodies coupled with horseradish peroxidase (Biorad, 1/50,000). The membranes were developed by chemoluminescence using ECL Advance (Amersham biosciences) and a CCD imager (FluorSMax, Biorad). Target protein concentrations were determined by fitting the sample signal values on these curves to protein standard curves.

### Remote Sensing data

The 2006 annual average vertical attenuation coefficients at 490 nm (k_490_) were obtained from the MODIS project [42].

## Supporting Information

Data S1 Parameterisation of photosystem II photoinactivation
(0.04 MB DOC)

Figure S1 Exponential decays of PSII capacity in lincomycin treated cultures of the five picocyanobacteria. In contrast to Figure 2, the photoinhibitory photon dose was calculated as coming through the photosynthetic antenna, by multiplying E×time×σPSII for the X-axis. Note the greater scatter among species in this plot compared to Figure 2.
(0.19 MB TIF)

Figure S2 The initial level and subsequent variations in the core subunit D1 of Photosystem II among the five marine cyanobacteria during exposure to a high light episode and recovery. D1 protein was determined by quantitative immunoblotting in cultures treated (closed) or not (open) with the protein synthesis inhibitor lincomycin to block photosystem II repair (n = 4, ±1 s.e.). The high irradiance episode is delineated by dotted lines. Note that in the absence of repair, *Synechococcus* RSS9917 was able to degrade and clear D1 proteins from photoinactivated photosystems II (A) as seen by the rapid 70% decrease in D1 content in cultures treated with lincomycin. In contrast, *Prochlorococcus* SS120 appeared to have limited 30% clearance of D1 protein during the high light episode (E), in spite of suffering significant photoinactivation of PSII (Figure 1E).
(0.30 MB TIF)
