# Supplementary material for: Light Variability Illuminates Niche-Partitioning among Marine Picocyanobacteria
Source: PLoS One. 2007 Dec 19;2(12):e1341. doi: 10.1371/journal.pone.0001341 (PMC2129112; doi:10.1371/journal.pone.0001341)
Supplement: Data S1 — Parameterisation of photosystem II photoinactivation (0.04 MB DOC) [file pone.0001341.s001.doc]

**Supplementary Data 1: Parameterisation of photosystem II photoinactivation**

Previous studies have analyzed photoinactivation in terms of rate constants, quantum yields (Fig. S1) or biological weighting functions [23-25,29,30,41,43-46]. We, however, used target theory [28] to parameterize the PSII photoinactivation rate as:

PSII photoinactivation rate = E • │σ_i_│

where E is the scalar irradiance (photons nm^-2^ s^-1^) and │σ_i_│ (nm^2^) is the magnitude of the effective target cross section for PSII photoinactivation (functional PSII lost s^-1^). In this formulation σ_i_ can be interpreted as the product of the absorbance cross section (nm²) for the target which absorbs the inactivating photon, and a quantum yield (dimensionless) for the photoinactivation event per absorbed photon (see also Supplementary Data 2). Thus, σ_i_ carries units of nm² but its nominal size does not reflect a physical size of the absorbing target. We estimated σ_i_ for blue light as the exponential decay of PSII function plotted versus cumulative photon dose nm^-2^ (Fig. 2), and found comparable values of σ_i_ for across the five picocyanobacteria species in spite of their diverse antenna structures and ecophysiological properties .The ratio σ_PSII_ :│σ_i_│then estimates the relative probability of photosystem II photochemistry versus photoinactivation, for a given spectral quality of light.

Using an alternate model for photoinactivation we attempted to fit the PSII photoinactivation rate as a function of the photon dose per PSII, estimated as:

PSII photoinactivation rate = E • σ_PSII_ • Φ_i_

where E is again the scalar irradiance (photons nm^-2^ s^-1^), σ_PSII_ is the effective absorbance cross section serving PSII photochemistry (nm^2^ PSII^-1^) and Φ_i_ is a quantum yield for photoinactivation relative to the photons delivered to PSII through the photosynthetic antenna [23]. For this parameterization we estimated Φ_i_ as the exponential decay of PSII function plotted versus cumulative photon dose PSII^-1^ (Fig. S1), with σ_PSII_ determined for each time point using flash fluorescence induction profiles. For blue light treatments of picocyanobacteria, this parameterization generated a 2.3 fold range of estimated Φ_i_ quantum yields across the five species (Fig. S1), contrasting with the tightly clustered values for σ_i_ among the strains (compare with Fig. 2). Furthermore, the Φ_i_ model predicts increasing rates of photoinactivation with increasing σ_PSII_, but the measured rates of photoinactivation across the cyanobacterial strains showed only a weak correlation with their σ_PSII_ effective absorbance cross sections serving PSII photochemistry (Table 1). Therefore, photoinactivation by typical PAR in marine picocyanobacteria is more coherently and directly explicable through the simpler model of photoinactivation resulting from an initial event separate from the photosynthetic antenna [27,30]), which we parameterize herein as σ_i_.

The target size parameterization we use allows ready estimation of the rate of primary photoinactivation as E • σ_i_. Furthermore, *R_PSII_* can now be readily estimated, without inhibitor incubations, through a comparison of the underlying primary photoinactivation, E • σ_i_, to the net change in photosystem II function over time, monitored using the widely available PSII fluorescence quantum yield (F_V_/F_M_).
