# Supplementary figures and images for: Light Variability Illuminates Niche-Partitioning among Marine Picocyanobacteria
Source: PLoS One. 2007 Dec 19;2(12):e1341. doi: 10.1371/journal.pone.0001341 (PMC2129112; doi:10.1371/journal.pone.0001341)

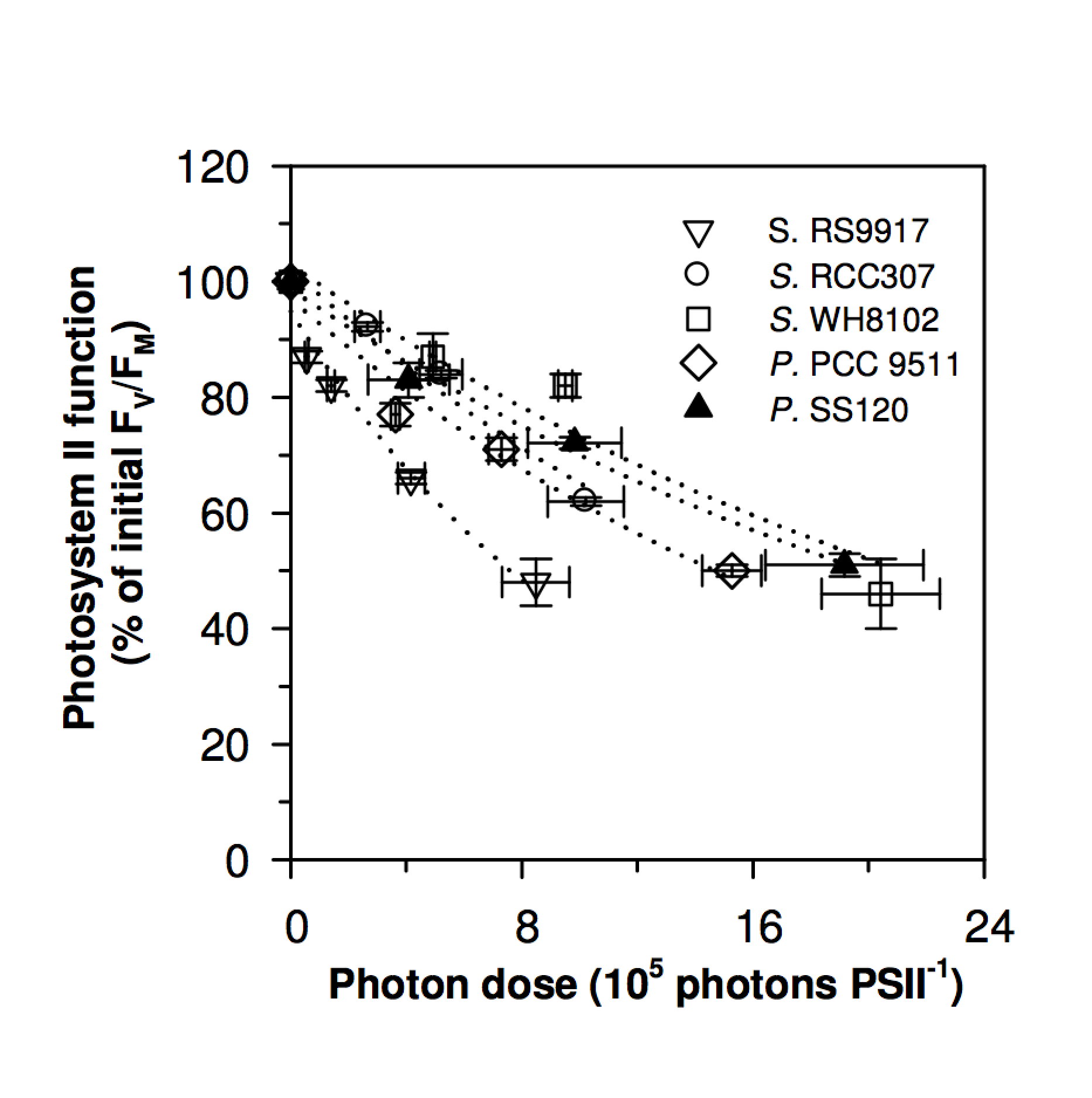

Supplement: Figure S1 — Exponential decays of PSII capacity in lincomycin treated cultures of the five picocyanobacteria. In contrast to Figure 2, the photoinhibitory photon dose was calculated as coming through the photosynthetic antenna, by multiplying E×time×σPSII for the X-axis. Note the greater scatter among species in this plot compared to Figure 2. (0.19 MB TIF) [file pone.0001341.s002.tif]

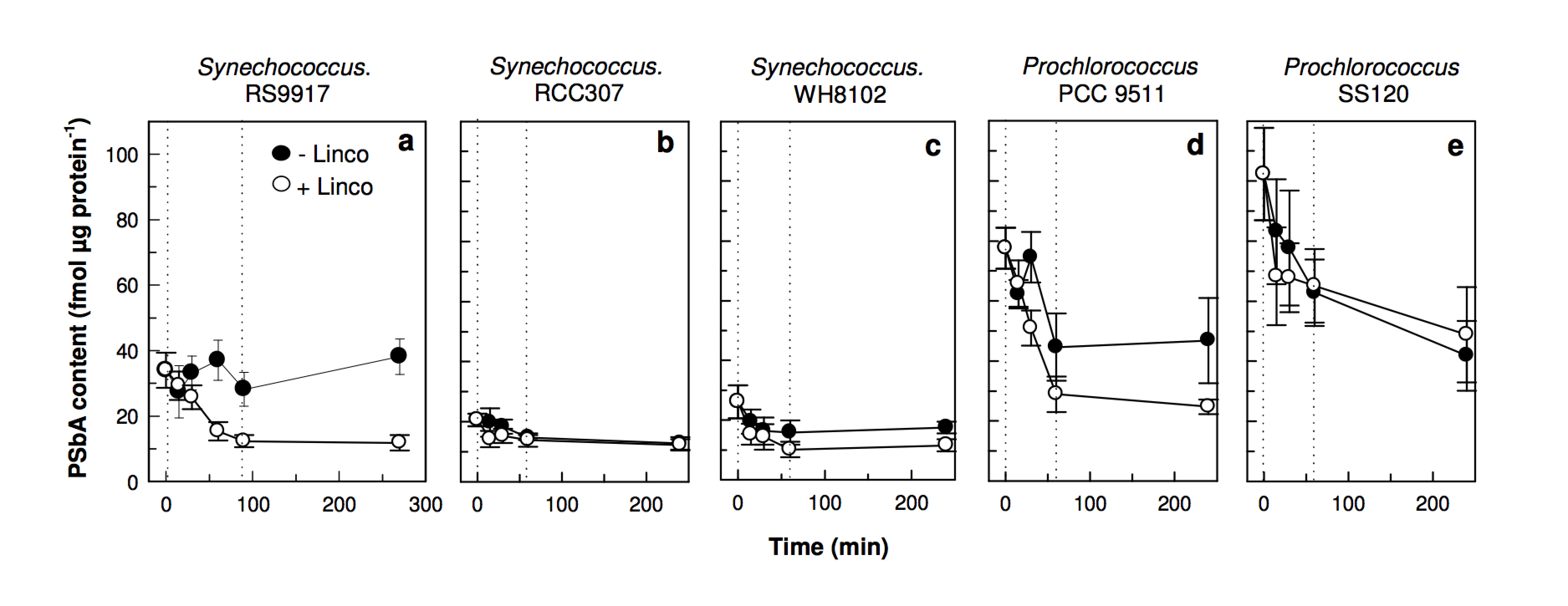

Supplement: Figure S2 — The initial level and subsequent variations in the core subunit D1 of Photosystem II among the five marine cyanobacteria during exposure to a high light episode and recovery. D1 protein was determined by quantitative immunoblotting in cultures treated (closed) or not (open) with the protein synthesis inhibitor lincomycin to block photosystem II repair (n = 4, ±1 s.e.). The high irradiance episode is delineated by dotted lines. Note that in the absence of repair, Synechococcus RSS9917 was able to degrade and clear D1 proteins from photoinactivated photosystems II (A) as seen by the rapid 70% decrease in D1 content in cultures treated with lincomycin. In contrast, Prochlorococcus SS120 appeared to have limited 30% clearance of D1 protein during the high light episode (E), in spite of suffering significant photoinactivation of PSII (Figure 1E). (0.30 MB TIF) [file pone.0001341.s003.tif]
